# Design of an *Arabidopsis thaliana* reporter line to detect heat-sensing and signaling mutants

**DOI:** 10.1186/s13007-023-01033-x

**Published:** 2023-06-08

**Authors:** Anthony Guihur, Baptiste Bourgine, Mathieu E. Rebeaud, Pierre Goloubinoff

**Affiliations:** 1 Department of Plant Molecular Biology, Faculty of Biology and Medicine, University of Lausanne, 1015 Lausanne, Switzerland; 2 Institute of Physics, School of Basic Sciences, École Polytechnique Fédérale de Lausanne (EPFL), 1015 Lausanne, Switzerland

**Keywords:** Global warming, Nanoluciferase, d-Amino acid oxidase, Heat-shock proteins, Heat-stress, HSP20, HSP101, HSP17.3b, Chaperones, Heat-inducible promoter

## Abstract

**Background:**

Global warming is a major challenge for plant survival and growth. Understanding the molecular mechanisms by which higher plants sense and adapt to upsurges in the ambient temperature is essential for developing strategies to enhance plant tolerance to heat stress. Here, we designed a heat-responsive *Arabidopsis thaliana* reporter line that allows an in-depth investigation of the mechanisms underlying the accumulation of protective heat-shock proteins (HSPs) in response to high temperature.

**Methods:**

A transgenic *Arabidopsis thaliana* reporter line named “Heat-Inducible Bioluminescence And Toxicity” (HIBAT) was designed to express from a conditional heat-inducible promoter, a fusion gene encoding for nanoluciferase and d-amino acid oxidase, whose expression is toxic in the presence of d-valine. HIBAT seedlings were exposed to different heat treatments in presence or absence of d-valine and analyzed for survival rate, bioluminescence and HSP gene expression.

**Results:**

Whereas at 22 °C, HIBAT seedlings grew unaffected by d-valine, and all survived iterative heat treatments without d-valine, 98% died following heat treatments on d-valine. The HSP17.3B promoter was highly specific to heat as it remained unresponsive to various plant hormones, Flagellin, H_2_O_2_, osmotic stress and high salt. RNAseq analysis of heat-treated HIBAT seedlings showed a strong correlation with expression profiles of two wild type lines, confirming that HIBAT does not significantly differ from its Col-0 parent. Using HIBAT, a forward genetic screen revealed candidate loss-of-function mutants, apparently defective either at accumulating HSPs at high temperature or at repressing HSP accumulation at non-heat-shock temperatures.

**Conclusion:**

HIBAT is a valuable candidate tool to identify *Arabidopsis* mutants defective in the response to high temperature stress. It opens new avenues for future research on the regulation of HSP expression and for understanding the mechanisms of plant acquired thermotolerance.

**Supplementary Information:**

The online version contains supplementary material available at 10.1186/s13007-023-01033-x.

## Background

Land plants are increasingly challenged by rising mean seasonal temperatures, leading to more frequent, lengthy, and extreme heat waves, severe droughts, and devastating fires. Excessive heat at the cellular level can negatively impact the structure and function of macromolecules, such as membranes and proteins, which are particularly thermo-labile [38]. Heat can form inactive protein aggregates with exposed hydrophobic residues that associate with other proteins and membranes [46]. Protein aggregate interactions with membranes can trigger the production of reactive oxygen species (ROS) that elicit apoptosis [37, 55].

Understanding the cellular and molecular mechanisms by which land plants, especially crops, can anticipate and respond to excessive temperatures, is crucial to address the agricultural challenges of global warming. Indeed, during the morning of a warm summer day, plants must detect an impending noxious heat stress and accumulate various protective heat-shock proteins (HSPs) and thermo- and ROS-protective metabolites, in order to withstand noxious temperatures at noon, and survive until evening [32, 33].

In plants, some heat-induced proteins (HSPs) are enzymes catalyzing the production of heat-protective and ROS-quenching metabolites. Whereas heat-accumulated metabolites, such as glycine betaine and trehalose, can stabilize native proteins and render them less thermolabile [18, 40], millimolar concentrations of metabolites can also reduce the heat-induced hyperfluidization and disruption of membranes [59]. In addition, many HSPs are molecular chaperones. Some heat-accumulating chaperones, such as the HSP20s, do not use ATP to reduce the aggregation of heat-labile proteins [2, 60, 74] and to prevent the disruption of hyper-fluidized membranes [20, 42]. Other conserved families of heat-induced core-chaperones, such as HSP70s, HSP100s, and HSP60s, function as ATP-fueled polypeptide unfolding enzymes [26, 32, 47, 77] that can convert inactive, potentially toxic protein aggregates into native functional proteins, even under heat-shock temperatures [4, 17, 23, 29, 50, 73].

In higher plants, cyclic nucleotide-gated channels CNGC2 and CNGC4, which are members of a family of 20 different stress-responsive membrane-embedded ion channels [41], act as primary heat sensors [24, 65]. Analogous to the animal heat sensor TRPV1, that was distinguished in 2022 by a Nobel prize to David Julius [10, 32], CNGC2-CNGC4 hetero-tetrameric channels respond to heat-induced increments in the fluidity of the surrounding plasma membrane, by mediating the transient entry of extracellular Ca^2+^. This initiates a specific signal, involving calmodulin and kinases that hyper-phosphorylates heat-shock transcription factor 1 (HSF1) in the cytosol and induce bound inhibitory HSP70s and HSP90s to dissociate from the inactive HSF1 monomers [56]. Ultimately, this signal, generally called the heat-shock response (HSR), leads to the massive accumulation of thermo-protective HSPs [31, 32].

Here, we designed a stable *Arabidopsis thaliana* reporter line called HIBAT, expressing a fusion gene encoding nanoluciferase (nLUC) and d-amino acid oxidase (DAO) under the control of a recombinant conditional heat-inducible promoter (HSP17.3B) from soybean. Whereas heat-treated HIBAT plants grown without d-Valine produced a strong bioluminescence signal, heat treatments killed most of the plants grown on d-Valine. HIBAT therefore possessed two unique properties for prospective genetic screens, aiming to identify mutants defective in heat-signaling and in the repression mechanism of the plant HSR at low temperature.

## Results

### Design and characterization of HIBAT

A 511-nucleotide fragment, from the soybean heat-inducible conditional promoter HSP17.3B (Additional file 1: Tab 12), has been previously used to design a heat-inducible GUS reporter in *Physcomitrium patens* [63]. Here, using the same promoter, we aimed to generate a heat-inducible reporter line in the vascular plant *Arabidopsis thaliana* that can conditionally express a fusion gene encoding in N-terminal for nanoluciferase (nLUC), fused to d-Amino acid Oxidase (DAO) (Additional file 2: Fig. S1). The transformed plants were expected to produce, following a heat shock, a strong and stable bioluminescence in the presence of furimazine [21], and generate deadly toxic reactive oxygen species in the presence of d-valine, thereby selectively killing plants with an effective heat shock response [28].

*Arabidopsis thaliana* Col-0 plants were transformed using the floral dip method (see “Materials and Methods”) and homozygous T2 transformants were selected for their ability to produce, in the presence of added furimazine, a strong luminescence signal following one hour heat-treatment at 38 °C. Since any leaky expression of nLUC-DAO on d-valine at 22 °C was expected to be toxic, our selection was further narrowed down to transformants that were totally devoid of nLUC expression at 22 °C. Among them, HIBAT was found to contain a single copy of the transgene, located in an intergenic region of the genome, thereby minimizing potential adverse effects on neighboring essential genes. Whole plant DNA sequencing of HIBAT confirmed a single T-DNA insertion with the transgene at position 26180497 on chromosome 1 (Additional file 3: Fig. S2). The insertion site was 253 nucleotides downstream of a putative DNA polymerase pseudogene (AT1G69590) and 834 nucleotides upstream of AT1G69600, an expressed gene encoding a zinc finger homeodomain protein.

### HIBAT is similar to parental Col-0

At non-heat-shock temperatures (22 °C), both HIBAT and Col-0 *Arabidopsis thaliana* exhibited comparable seed germination percentages, root elongation rates, and fresh weight gains, indicating that the transgene did not negatively impact plant growth and development (Additional file 4: Fig. S3). However, the cotyledons of HIBAT’s seedlings, but not the true leaves, displayed a mild decrease of acquired thermotolerance and most turned white, compared to the cotyledons of Col-0 seedlings that stayed green (Additional file 5: Fig. S4). We nevertheless posited that such a mild heat-sensitive phenotype of HIBAT’s cotyledons, would not affect our ability to screen for HSR-defective seedlings with 6–8 true leaves. HIBAT was thus retained for its advantageous characteristics of having a single copy insertion of the transgene, not in a protein-coding gene, that could tightly repress nLUC-DAO expression at 22 °C, while massively accumulating it at 36 °C.

### Heat-induced expression of nLUC-DAO bioluminescence in *Arabidopsis* seedlings.

**Fig. 1:**
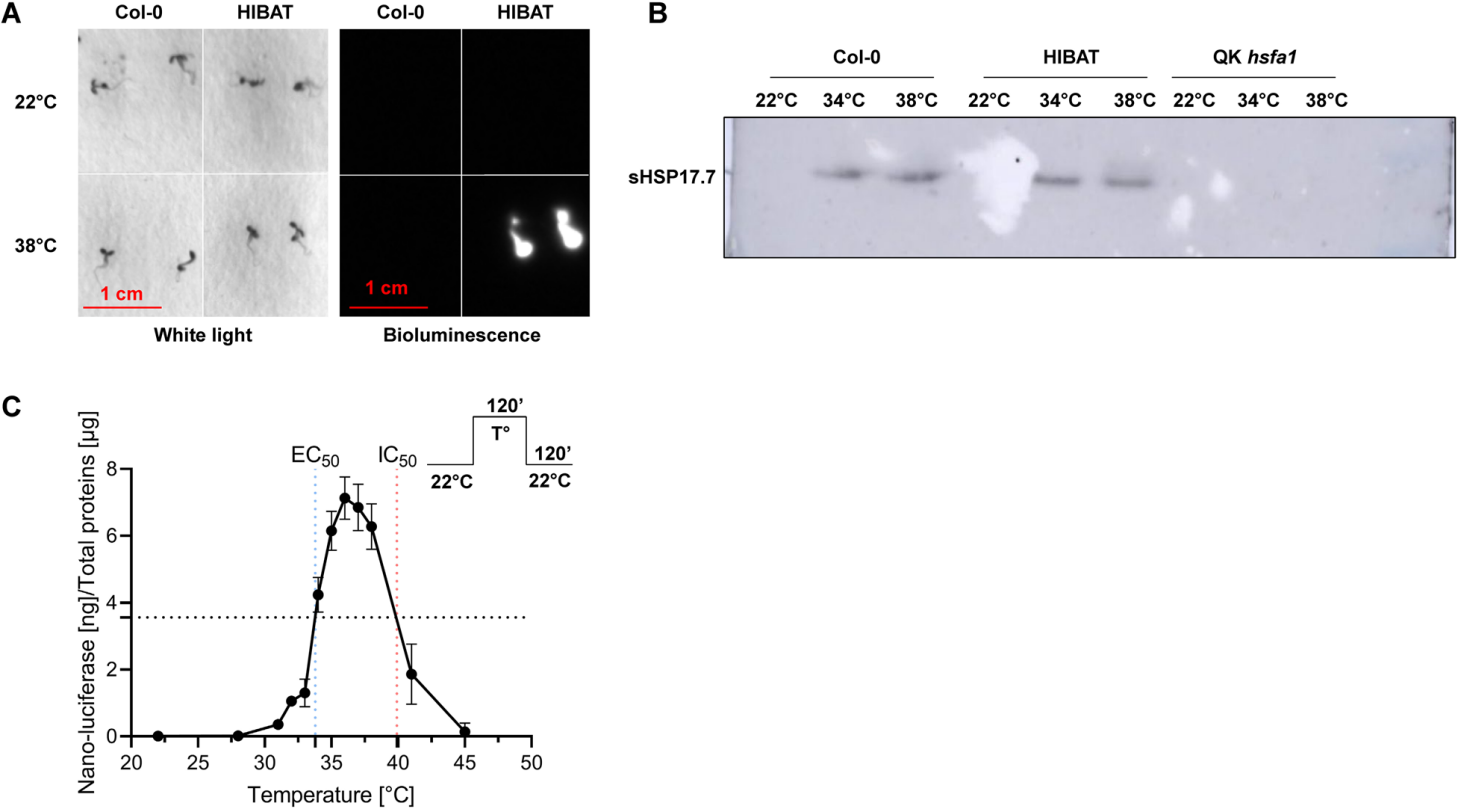
Heat-induced expression of nLUC-DAO bioluminescence. **A** Two-week-old HIBAT seedlings, grown at 22 °C, were subjected to a 2-h heat treatment at 36 °C and subsequently sprayed with a 1/100 dilution of furimazine. Images were captured after a 2-h recovery period at 22 °C. **B** Immunoblot analysis was performed to detect sHSP17.7 in Col-0, HIBAT, and a quadruple hsfa1 knockout (KO) mutant. Leaves were exposed to 22 °C, 34 °C, or 38 °C for 2 h, followed by a 2-h recovery at 22 °C. **C** The expression of the heat-inducible nLUC-DAO was assessed in HIBAT seedlings exposed to different temperatures for 2 h, followed by a 1-h recovery at 22 °C. The concentration of expressed nLUC-DAO proteins was quantified and compared to a calibration curve using purified nLUC. The results are presented as means ± standard deviation (S.D.) with a sample size of n = 8

To assess the heat-inducible expression profile of the nLUC-DAO transgene, HIBAT and Col-0 seedlings were exposed two-hours to a range of temperatures, between 22 °C and 38 °C. This was followed by a two-hour period at 22 °C to facilitate the completion of HSP synthesis. Seedlings were subsequently sprayed with a 1/100 furimazine solution. In the absence of prior heat shock, neither HIBAT nor Col-0 seedlings emitted light. In contrast, following HS, only HIBAT seedlings displayed strong bioluminescence in a simple chemiluminescence detection device (Fig. 1A).

The reliance of the light signal on HSP17.3B was verified through immunoblots and extended to other endogenous *Arabidopsis* HSPs. At 22 °C, endogenous *Arabidopsis* sHSP17.7 and HSP101 were virtually undetectable by immunoblots in both HIBAT and Col-0 seedlings. Following heat treatments at 34 °C and 38 °C, both sHSP17.7 and HSP101 were strongly accumulated in both Col-0 and HIBAT, but not in the quadruple *hsfa1* knockout *Arabidopsis* mutant that was used as a negative control (Fig. 1B) [49]. This was accompanied with a substantial accumulation of nLUC-DAO exclusively in HIBAT, as determined by nanoluciferase activity in total protein extracts (Additional file 6: Fig. S5). These results demonstrate that the transgene-encoded nanoluciferase activity in HIBAT can serve as a faithful reporter for heat-induced expression levels of endogenous HSPs, which are encoded in other regions of the plant genome, distally from the transgene.

To determine the temperature-dependent activation profile of the recombinant HSP17.3B promoter, nanoluciferase activity was measured in soluble extracts from HIBAT seedlings following various heat treatments between 22 °C and 45 °C. Below 28 °C, HSP17.3B-mediated expression of nLUC-DAO was tightly repressed. Above 28 °C, expression levels strongly increased, with half-maximal expression at 33.8 °C and a maximal expression at 36 °C. Above 36 °C, nanoluciferase overexpression declined with an IC_50_ at 40 °C (Fig. 1C). Since nanoluciferase is highly thermostable (Tm = 60 °C, Promega), this decline is more likely due to the heat-impairment of the protein synthesis machinery than to the heat-denaturation of nLUC per se.

### Impact of d-Valine on HIBAT survival at 22 °C and following heat-treatments

**Fig. 2:**
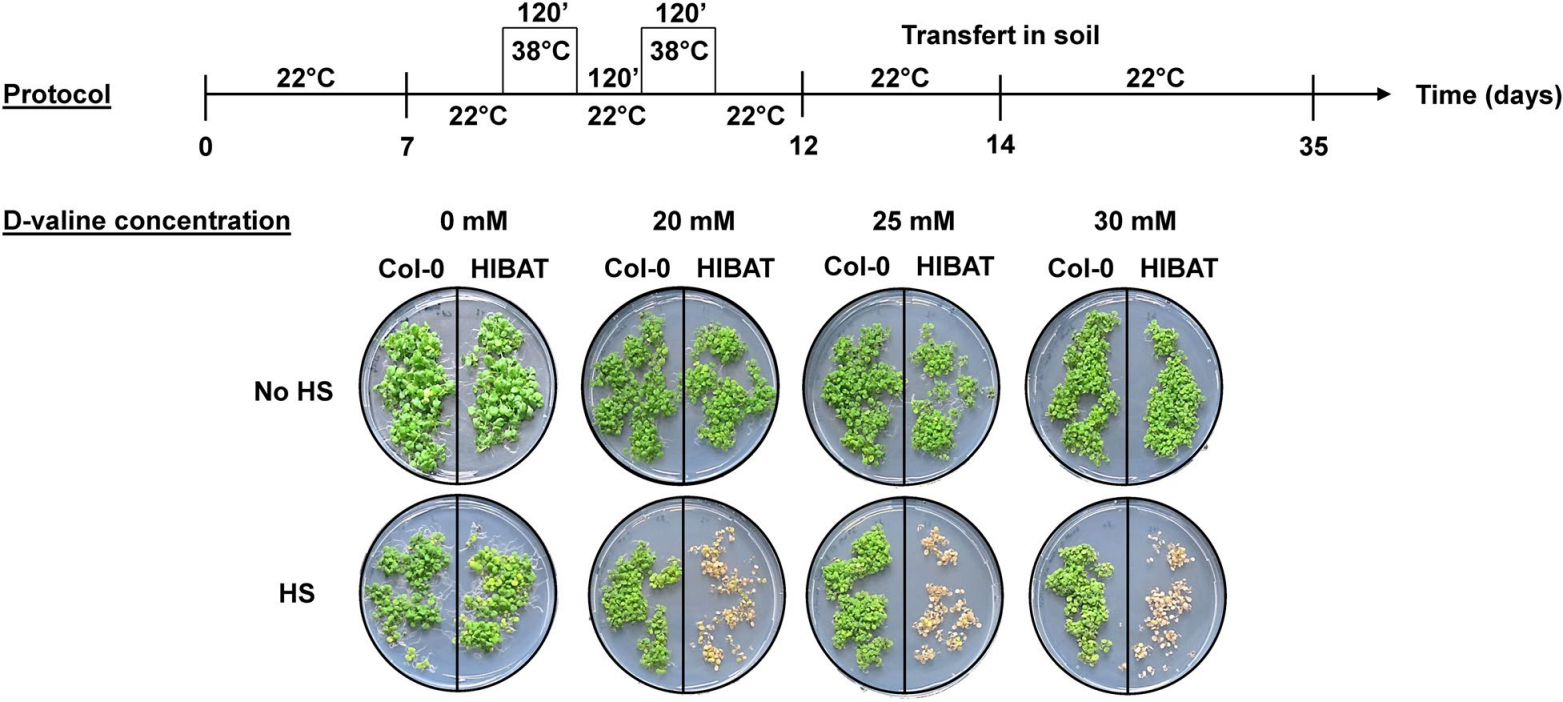
Two treatments at 38 °C in the presence of d-valine exhibit selective toxicity towards HIBAT seedlings compared to Col-0 seedlings. Above: Experimental protocol involving two-week-old Col-0 and HIBAT seedlings cultivated at 22 °C in the presence of increasing concentrations of d-valine, followed by two consecutive 120-min heat treatments at 38 °C, with a 120-min interval at 22 °C. Below: Representative images of Col-0 and HIBAT seedling lawns grown on Petri dishes containing 0 mM, 20 mM, 25 mM, and 30 mM d-valine, without heat treatment (no HS) or following the two heat treatments (HS). HIBAT seedling mortality was observed six days after the heat treatments

At 22 °C, both Col-0 and HIBAT seedlings grew undisturbed by the presence of up to 30 mM d-valine in the growth medium (Fig. 2). After two heat treatments, each of 120 min at 38 °C, separated by a 2-h at 22 °C, the majority of HIBAT seedlings grown on 25 mM d-valine perished within several days, while all wild-type Col-0 seedlings continued to grow and thrive. This demonstrates that the expression of nLUC-DAO, which is toxic on d-valine, is tightly repressed in HIBAT at 22 °C. Moreover, following a heat-treatment without d-valine, which is not lethal per se for both Col-0 and HIBAT, the amount of heat-accumulated nLUC-DAO in HIBAT was sufficient to generate on d-valine, enough toxic chemicals (keto-acid-3-methyl-2 oxobutanoate, NH3, and H_2_O_2_) to kill 98% of the seedlings (Fig. 2B, Additional file 6: Fig. S5). Consequently, HIBAT contained the necessary new characteristics for a specific *Arabidopsis* reporter line to conduct genetic screens aiming at identifying loss-of-function heat-sensing and heat-signaling mutants. These mutants were anticipated to withstand heat treatments in the presence of d-valine, and loss-of-HSP repression mutants were expected, in the absence of d-valine, to emit low levels of luminescence and HSPs at low temperatures.

### nLUC-DAO expression is heat-specific

**Fig. 3:**
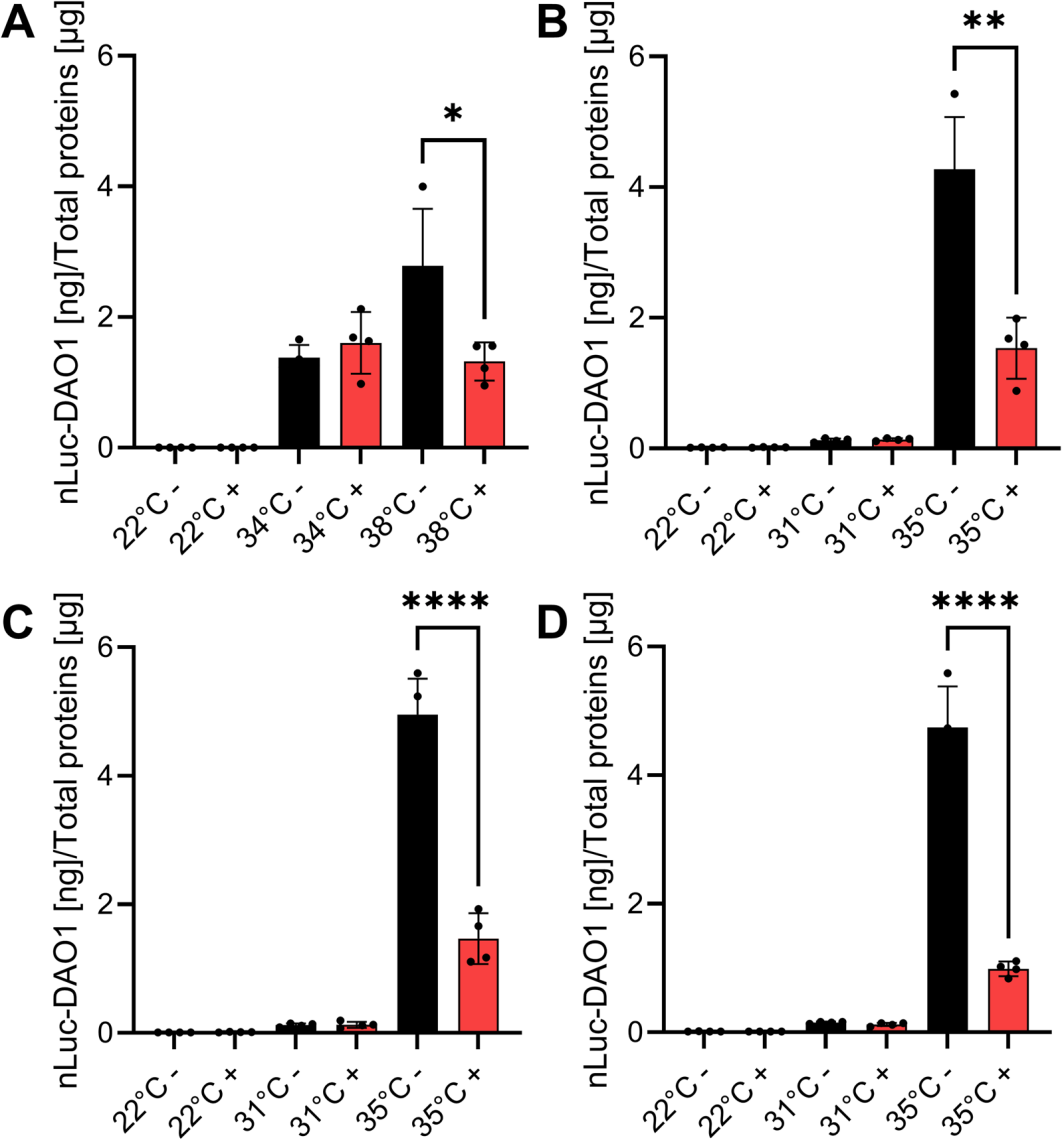
Effect of chemical compounds on the expression of nLUC-DAO1 at different temperatures. **A** Hydrogen peroxide (H_2_O_2_). **B** Mannitol. **C** Sodium chloride (NaCl). **D** Flagellin (FLG22). On day 12, twenty-two seedlings were exposed to either 250 µM H_2_O_2_, 300 mM mannitol, 150 mM NaCl, or 1 mM FLG22 for 5 h at 22 °C, followed by 2 h at the indicated temperatures and then 2 h at 22 °C. The absence of H_2_O_2_, mannitol, NaCl, and FLG22 is represented by the black bars, while their presence is indicated by the red bars. Statistical significance was determined using the Student's t-test (* P < 0.05, ** P < 0.01, *** P < 0.001, **** P < 0.0001, NS, Not Significant). The data are presented as means ± standard deviation (n = 4)

**Fig. 4:**
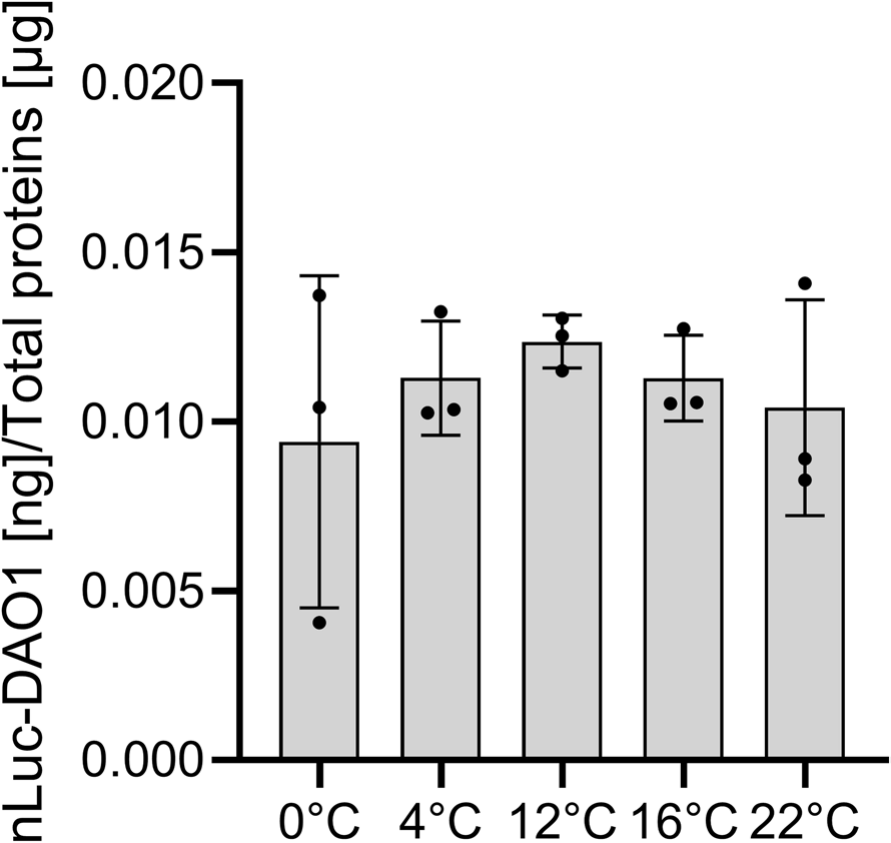
Effect of chilling and cold-shock on the expression of nLUC-DAO1 in HIBAT**.** On day 12, seedlings were subjected to different temperature treatments: cold shock (0 °C), chilling (4 °C to 16 °C), or maintained at room temperature (22 °C) for a duration of 2 h. Following the treatments, measurements were taken from cell extracts after 2 h of post-treatment at 22 °C. The data are presented as means ± standard deviation (n = 4). Statistical analysis using the Student's t-test showed no significant difference at 22 °C compared to all other conditions

**Fig. 5:**
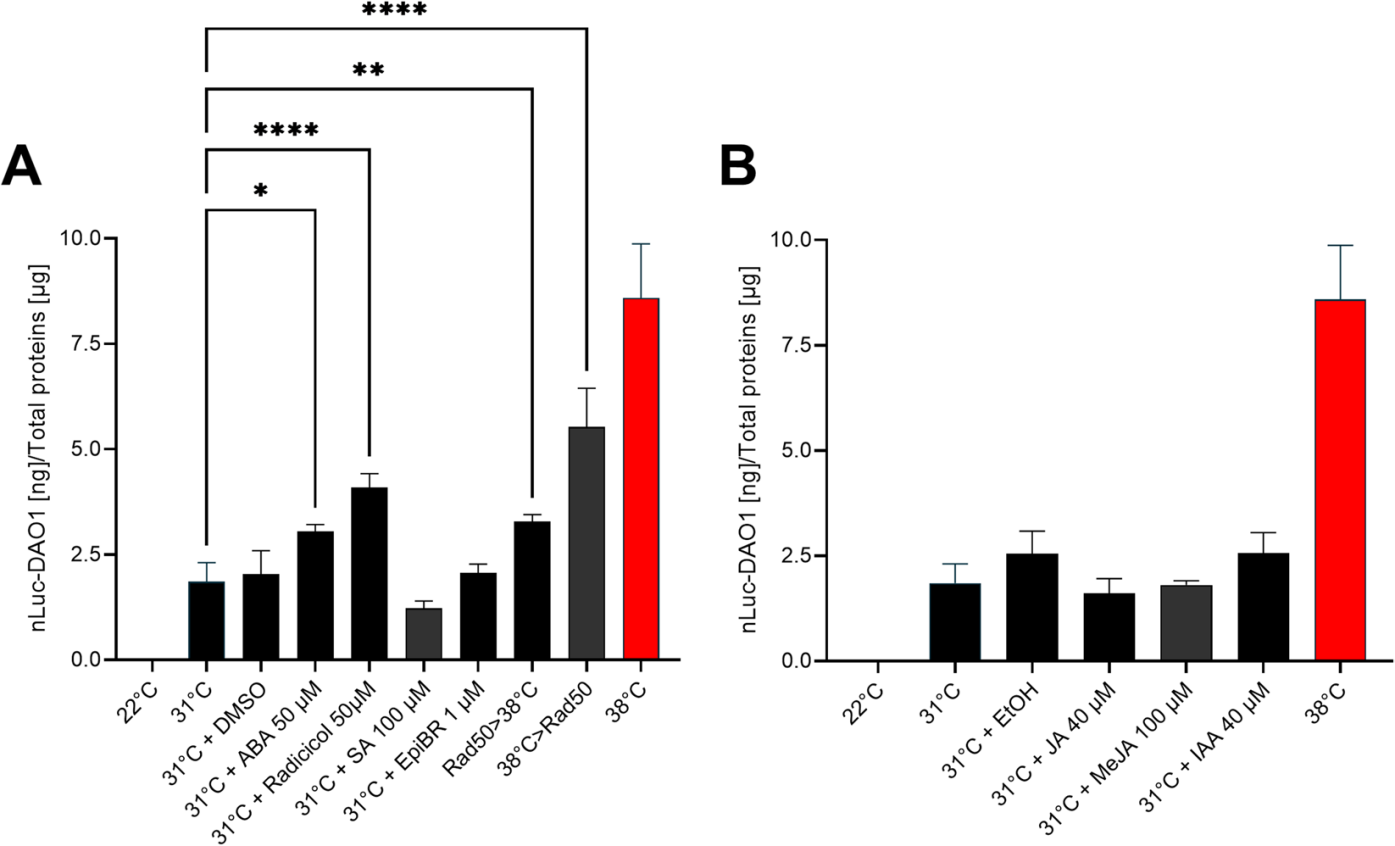
Effect of phytohormones on the expression of nLUC-DAO. On day 12, seedlings were subjected to different treatments: a 2-h heat shock at 38 °C, exposure to various phytohormones for 4 h at 31 °C, or no treatment at 22 °C. **A** Differential expression of the transgene in the presence of DMSO (mock), 50 µM ABA, 50 µM Radicicol, 100 µM salicylic acid (SA), 1 µM Epibrassinolide (EpiBR), 50 µM Radicicol at 22 °C for 2 h followed by heat shock at 38 °C for 2 h, and 2 h at 38 °C and then addition of 50 µM Radicicol for 2 h. **B** Differential expression of the transgene in the presence of ethanol (EtOH, mock), 40 µM Jasmonic Acid (JA), 100 µM Methyl-Jasmonate (JA), or 40 µM Indole-3-acetic acid (IAA, auxin). Statistical analysis using ordinary one-way ANOVA was performed, and asterisks indicate statistically significant differences (* P < 0.05, ** P < 0.01, *** P < 0.001, **** P < 0.0001, NS, Not Significant)

To screen for mutants affected in heat-sensing and heat-signaling, it was essential that the recombinant conditional HSP17.3B promoter would be specific for heat and remain unresponsive to other stressors. 12-day-old HIBAT seedlings were exposed for 5 h, either to 250 µM H_2_O_2_, 150 mM NaCl, 300 mM mannitol, or 1 mM FLG22, at various indicated temperatures and nanoluciferase expression levels were measured in seedling’s extracts (Fig. 3). In general, other stressors did not induce nLUC at 22 °C and mildly affected the maximal heat-induced accumulation at 35 °C and/or 38 °C: externally applied H_2_O_2_ showed a very minor activatory effect at low temperature that was, however, a thousand times lower than the maximal level of accumulated nLUC-DAO following 2 h at 36 °C. H_2_O_2_ mildly affected heat-induced nLUC-DAO expression (at 38 °C, Fig. 3A). Osmotic stress with mannitol did not have an effect at 22 °C, but it significantly affected the heat-induced accumulation of nLUC-DAO (Fig. 3B). NaCl stress had no activating effect at low temperatures, but it reduced nLUC-DAO levels at 35 °C (Fig. 3C). Additionally, flagellin FLG22 did not cause measurable effects at 22 °C, but it reduced heat-induced nLUC-DAO accumulation at 35 °C (Fig. 3D). Cooling and chilling at 0 °C for 5 h had no influence on nLUC expression (Fig. 4). Likewise, external application of various concentrations of Indole-3-acetic acid, Jasmonate, Methyl-Jasmonate, Epibrassinolide, and Salicylic acid did not affect transgene expression at 31 °C. Only Abscisic acid had a minimal activation effect (Fig. 5A, B). The HSP90 inhibitor radicicol (50 µM) had no effect at 22 °C, and a minor, yet significant, co-activatory effect at 31 °C (Fig. 5A). This is consistent with reports in *Arabidopsis* [82], animal, yeast and moss cells, whereby the specific binding of HSP90 inhibitors, such as radicicol, can cause the dissociation from HSP90s of bound clients, such as the inactive HSF1 the cytosol [57]. However, we did not observe in HIBAT any mild isothermal HSR activation by radicicol at 22 °C. Even a 2-h preincubation at 22 °C with 50 µM radicicol, neither enhanced, nor significantly reduce the plant's ability to respond to a subsequent heat shock (37 °C). The accumulation of heat-induced nLUC-DAO was nearly identical with or without radicicol preincubation (Fig. 5A). This suggests that the presumed radicicol-induced dissociation of bound HSP90s from inactive HSF1, does not suffice to induce an isothermal activation of the HSR, as generally though [36].

### Profile of HIBAT gene expression following heat or d-valine treatments.

We next examined the heat-induced mRNA expression profile of HIBAT, compared to the wild-type Col-0 parental strain. RNA-Seq analysis of HIBAT seedlings grown at 22 °C, with or without a 40-min 38 °C heat treatment, identified 25,000 transcripts, of which 2,668 were significantly upregulated, resulting in a net mRNA gain of 23% (230,000 transcripts per million, TPMs). In contrast, 2159 transcripts were significantly degraded during short the heat treatment, resulting in a net loss of approximately 28,000 TPMs. Many of the most heat-degraded transcripts encoded for chloroplast-imported proteins. The analysis excluded about 200,000 TPMs from the heat-degraded transcripts because of their excessive p-values, due to the variation in their relative degraded amounts between samples. Less variation was observed among the heat-accumulated mRNA and more HSP genes were retained as significantly over-expressed.

**Fig. 6:**
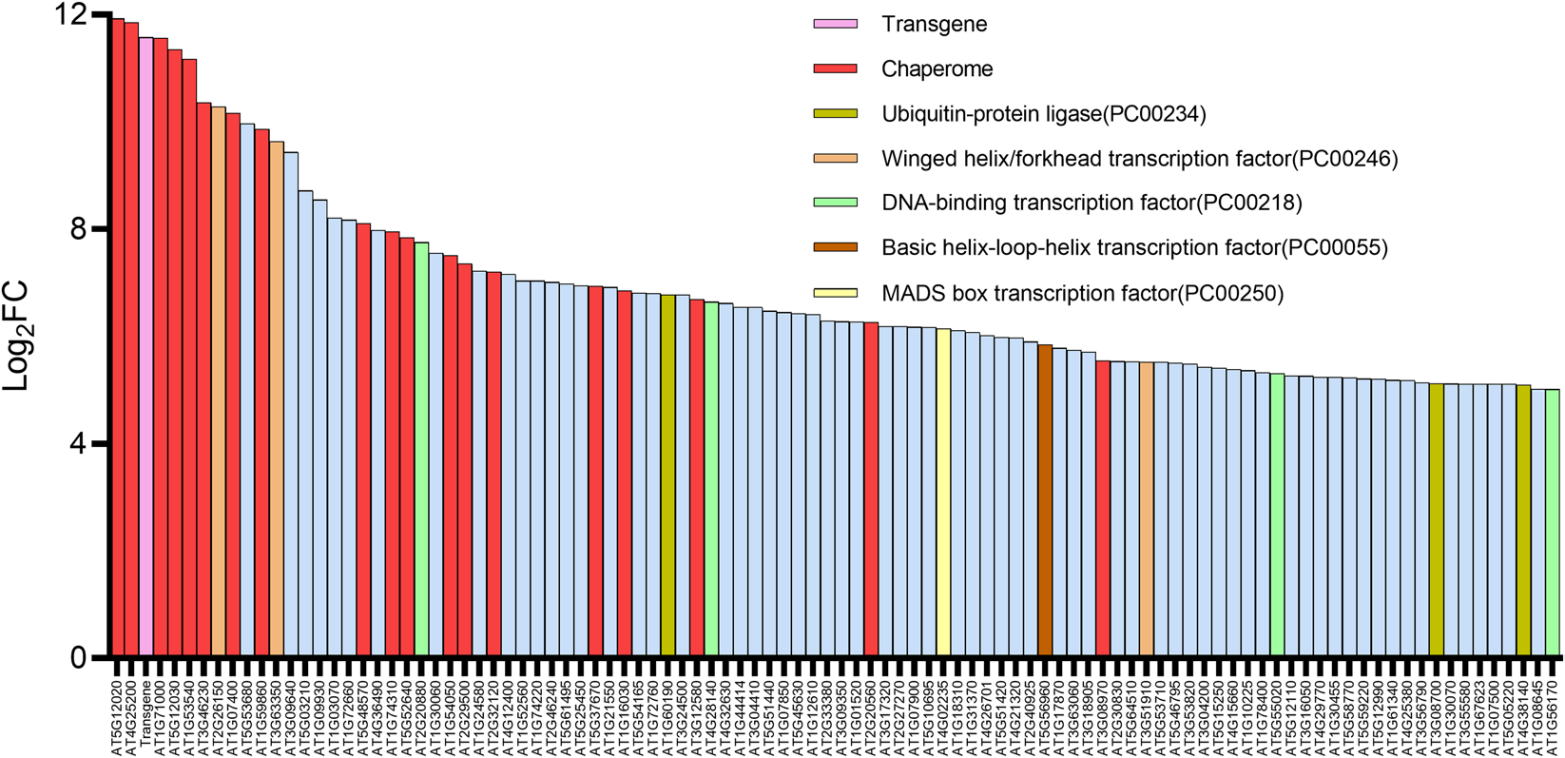
Identification of the top 100 differentially expressed genes in response to heat stress in Arabidopsis thaliana HIBAT seedlings. The heat-induced genes are ranked based on Log2FC (Fold-Change) values, which represent the fold increase in Transcripts per million (TPM) following a 40-min exposure to 38 °C, compared to the TPM in untreated 14-day-old HIBAT seedlings at 22 °C. The genes are categorized into specific functional groups: Transgene insertion in HIBAT (highlighted in pink), chaperones and cochaperones (chaperome) (highlighted in red), Ubiquitin ligases (highlighted in acid), heat shock transcription factors (HSFs) (highlighted in orange), DNA-binding transcription factors (highlighted in green), helix-loop-helix transcription factors (highlighted in brown), MADS-box transcription factors (highlighted in yellow), and other genes (highlighted in blue). The assignment of gene families was determined using PANTHER gene analysis [72]

Of the 18 detected genes encoding for HSP20s, which are alpha-crystalline-domain containing chaperones [27], 12 were within the 100 most extensively heat-induced genes of the entire *Arabidopsis* genome (Additional file 1: Tab 2). Interestingly, these 12 HSP20 genes were virtually unexpressed at 22 °C. Other highly upregulated genes in response to heat also included several members of the HSP90, HSP100, HSP70, and HSP60 chaperone families, as well as their co-chaperones (collectively referred to as the chaperome) (Fig. 6).

The presence of 25 mM d-valine at 22 °C had only a minimal effect on the plant mRNA expression profile: it caused a significant, yet very mild accumulation of merely 31 transcripts, and significantly reduced only 78 transcripts (Additional file 1: Tab 3). Importantly, none were among the 100 most heat-induced or heat-degraded transcripts. Thus, RNAseq analysis confirmed that the presence of 25 mM d-valine in the growth medium has but minimal to insignificant effects on the plant physiology.

**Fig. 7:**
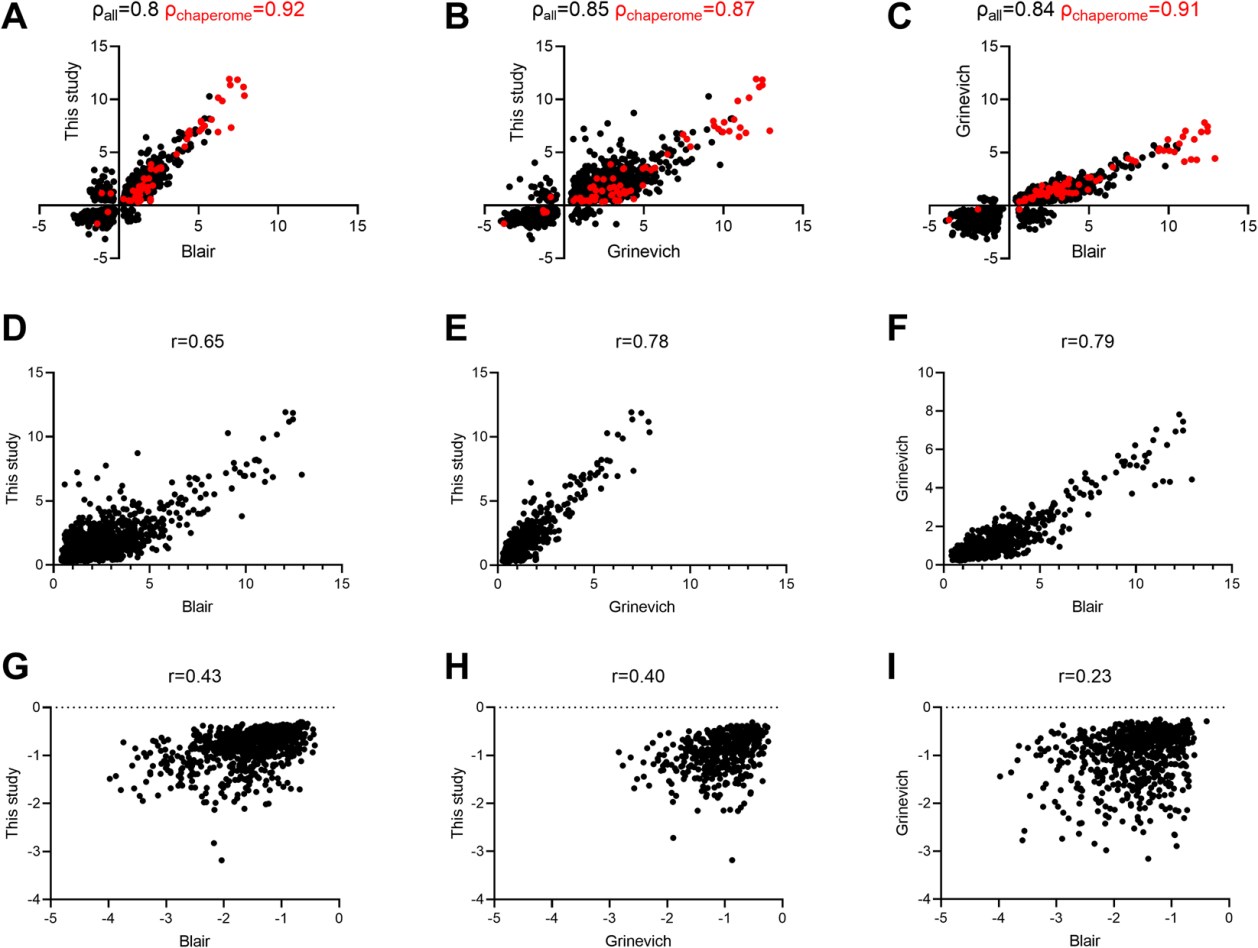
Scatter Plot showing a correlation in Log2 fold-change expression between two pairs of datasets**. A**: Correlation between this study and Blair et al. **B** Grinevich et al., and **C** between Blair and Grinevich. Spearman's rank correlation coefficient (ρ) is indicated in black for all genes and red for the chaperomes. **D**-**F**: All upregulated genes. **G**-**I**: All downregulated genes

RNAseq analysis from heat-treated HIBAT showed a strong correlation with two published studies using similar heat-treatments of Col-0 seedlings [7, 30] (Fig. 7). A correlation analysis between the Log_2_ Fold-change in heat-accumulated, versus unstressed transcripts, between the current HIBAT study and two published studies with Col-0 [7, 30] showed a high positive correlation for all the analyzed genes, with a Spearman correlation coefficient (*ρ)* of 0.8, 0.85 and 0.84, respectively (Fig. 7A, B, and C). When considering only chaperome genes (in red), the correlations were 0.87 and 0.92 (Fig. 7A, B, and C in red). This implies that heat shock response expression profiles of HIBAT and wild type Col-0 are very similar.

We next addressed the potential use of HIBAT as a tool to screen for loss-of heat-sensing and signaling mutants. EMS-treated HIBAT seedlings were grown at 22 °C in the presence of 20 mM d-Valine and exposed twice a day for 120 min at 38 °C interspersed with 2 h of recovery (Additional file 7: Fig. S6).

98% of the mutant HIBAT seedlings did not survive the iterative heat-treatment. Interestingly, many of the survivors were found to be also defective following heat-treatment at expressing both bioluminescence from the transgene and the endogenous HSP17.7 and HSP101. As an example, the five selected lines shown in Additional file 8: Fig. S7, which all survived the iterative heat-shocks on d-valine and were all defective in expressing heat-induced nLucDAO, all displayed after heat-treatment at least 50% less HSP17.7 and HSP101 than wild type HIBAT (Additional file 8: Fig. S7).

We also addressed the potential use of HIBAT as a tool in a genetic screen to identify genes involved in the repression of the HSR at low temperature. Hence, when ~ 3′800 EMS-mutagenized M2 seedlings of HIBAT grown at 22 °C in the absence of d-Valine were sprayed with furimazine, all as expected did not emit light at 22 °C, with the notable exception of two potential mutants (red circles, Additional file 9: Fig. S8A). One candidate mutant named “L5”, which displayed a strong basal bioluminescence in its cotyledons, was further analyzed at the rosette stage by bioluminescence (Additional file 9: Fig. S8B) and Western blot analysis and higher basal expression levels of the endogenous sHSP17.7 and HSP101 were found, compared to the parent wild type HIBAT (Additional file 9: Fig. S8C). The mutation(s) causing in L5 the apparent de-repression of the transgene and of sHSP17.7, HSP101 at low temperature, are currently under investigation.

Whereas candidate mutants still need to be thoroughly characterized, these preliminary results indicate that HIBAT can serve as a useful versatile tool to screen for loss-of heat-sensing and -signaling mutants at high temperatures, and for loss-of-HSR repression mutants at low temperatures.

## Discussion

### Characterization of HIBAT as a Heat Shock Reporter Line

To investigate the molecular mechanisms leading to the accumulation of HSPs in response to elevated temperatures, we generated the transgenic *Arabidopsis* reporter line HIBAT. It featured the heat-inducible HSP17.3B promoter derived from soybean [63, 66] driving the conditional expression of a fusion transgene composed of nano luciferase and d-amino acid oxidase (nLUC-DAO). Upon heat-induced expression of the transgene, the N-terminal domain of nanoluciferase (nLUC), HIBAT was found to generate a strong and stable bioluminescence signal. This signal correlated with the levels of endogenous heat-accumulated HSP20 proteins as well as of other gene families of HSPs (Additional file 1: Tab 2), while expression of the C-terminal DAO domain was found to be conditionally toxic. However, this toxicity occurred only in the presence of d-valine, which results in the production of harmful reactive oxygen species [58, 68, 71, 76]. Due to the presence of a single copy of the transgene, in a non-coding intergenic region, HIBAT did not express any leaky detectable nLUC-DAO and consequently it grew unaffected by d-Valine at 22 °C. In contrast, 98% of the HIBAT seedlings did not survive heat treatments on d-Valine (Fig. 2B, Additional file 6: Fig. S5). Heat-treatment (without d-valine) also accumulated endogenous HSP17.7 and HSP101 in heat-treated Col-0 and HIBAT seedlings, but not in quadruple *hsfa1* mutant that was used here as a control, confirming that it cannot produce HSPs in response to a heat-shock (Fig. 1B) [49].

In addition to physiological assays (Additional file 4: Fig. S3), RNA-seq analysis of untreated and heat-treated HIBAT seedlings also confirmed that, like in the wild-type Col-0, most cytosolic members of the HSP20 family are virtually unexpressed under basal growth temperature, and become dramatically overexpressed in response to a heat-shock [8, 31, 63]. This is suggesting that although HSP20s are essential for plant recovery from heat stress [54], their futile expression at low temperature may be, for unclear reasons, detrimental to unstressed plants [8, 31–33, 69, 81]. In contrast, other families of heat-induced chaperones, such as the HSP60s, HSP70s, HSP40s, HSP90s, and HSP100s, which have orthologs with high levels of constitutive expression at low temperature (e.g., chloroplastic HSP70, AT4G24280), are proportionally only mildly heat-induced. But this may be a misconception resulting from expressing the effect of a heat treatment as a log of a fold change of heat-accumulated TPMs divided by the ground temperature TPMs, rather than as their net difference (Additional file 1).

### The HSP17.3B promoter is highly specific to heat

Identical heat-treatments can produce much more HSP mRNAs in dehydrated plants, than in hydrated plants [13, 83]. Yet, for an *Arabidopsis* reporter line designed to screen for mutants specifically affected in plant heat-sensing and signaling, we sought for an expression system specific for heat, with minimal interference from other stressors. Indeed, the synthesis of the reporter protein in HIBAT was found to be specific to heat with no significant interferences observed for salt stress (NaCl) and osmotic stress (mannitol), although both are thought to mediate Ca^2+^- and H_2_O_2_-mediated cytosolic signals that also lead to HSPs accumulation [5, 39]. Pathogen-like stress (flagellin-22), and various phytohormones remained ineffective, except for a minor activatory effect by abscisic acid, confirming previous evidence that abscisic acid is necessary for the onset of plant thermotolerance [39, 70]. A similar very minor activatory effect was found for H_2_O_2_, which participates in the activation of HSFs and acts in various organisms as a secondary messenger, leading to HSP expression [11, 16, 53, 78]. We did not observe an effect of cooling and chilling [34], but this is not unexpected from more rigid plasma membranes, expected to freeze up more than activate the CNGC2/4 heat sensors.

### The effect of radicicol: implications for HSF1 activation

Concurring with previous reports in mosses and other organisms [36, 57], the minor accumulation of nLUC-DAO at 31 °C was co-activated by the HSP90 inhibitor radicicol (Fig. 5A). Yet, remarkably, this did not yield a maximal heat-shock response. This is suggesting that the heat-induced activation of hypophosphorylated HSF1 requires more than its mere dissociation from inhibitory HSP90s (and HSP70s), as generally believed [36]. Indeed, experimental evidence have shown that HSF1 activation does not take place in the absence of a specific Ca^2+^ entry-dependent signal originating in the periplasm through the CNGC2/4 channels in the plasma membrane. This heat-shock signal is central to the heat-induced hyperphosphorylation of HSF1 and the release of inhibitory chaperones, such as HSP90 and HSP70 [24, 64].

The transgene was inserted close to AT1G69600, encoding for ZFHD1. This member of the zinc finger homeodomain transcription factor family accumulates under drought, high salinity, and abscisic acid (ABA). Whereas the transgene expression was unresponsive to these stresses, the very low basal expression levels of ZFHD1, were nearly doubled in response to heat, suggesting a possible mild overlap between the two stresses.

The RNAseq analysis confirmed the primary importance of HSP20s in the plant response to heat stress, in line with previous observations [31]. Yet, aside from in vitro indications that HSP20s may generally prevent protein aggregation and possibly protect membranes from hyperfluidization [74], it is not clear what is the specific mechanism by which the heat accumulated HSP20s effectively contributes to the onset of plant acquired thermotolerance. Given that plant acquired thermotolerance also results from a prior heat-priming-induced repression of a proapoptotic signal, which would otherwise be deadly to the heat-shocked plants [24], it is tempting to speculate that heat-pre-accumulated HSP20s may contribute to plant acquired thermotolerance by repressing apoptotic signals otherwise deadly, during a noxious heat-stress, which, if they were to be constitutively expressed in unstressed plants, would in the long term affect cellular differentiation and tissue development.

It is worth noting many chaperome genes in the plant genome were either undetected, or did not become significantly heat-accumulated, or were heat-degraded, as observed in the case of AT3G04980, AT2G41000, AT3G12170, AT5G12430, AT5G06910, and AT2G33210. This confirms that chaperones and co-chaperones should not be misleadingly referred to as heat-shock proteins [25, 31].

### The potential use of HIBAT to identify HSR impaired mutants.

Our pilot investigation of HIBAT, as potential tool to screen for loss-of heat-sensing and signaling mutants has provided partial but nevertheless insightful findings: of the ~ 2% of the EMS-treated HIBAT seedlings that survived a protocol of iterative heat-stresses on d-Valine (Additional file 7: Fig. S6), were found to be also compromised in their response to heat treatment. They were defective not only at heat-inducing the expression of the transgene, but also the endogenous HSP17.7 and HSP101 encoded elsewhere in the genome. This suggests that future screens with HIBAT may potentially identify mutations in upstream sensors, mediators and master regulators of plant heat shock response in general (Additional file 8: Fig. S7).

Similarly, our pilot investigation also identified two candidate HIBAT mutants that abnormally expressed bioluminescence from the transgene at 22 °C (red circles, Additional file 9: Fig. S8), one of which, named "L5", that was also found by western blot to express higher levels of endogenous sHSP17.7 and HSP101, compared to the parental WT HIBAT (Additional file 9: Fig. S8). This preliminary finding indicates that a genetic screen with HIBAT can also be used to identify repressors of the HSR at low, non-heat-shock temperatures. A similar approach has already been successfully used using firefly luciferase expressed from a heat-conditional promoter of cytosolic HSP70. A disinhibited bioluminescence phenotype at 18 °C, led to the identification of a component of a chromatin remodeling complex, Arp6, to be a repressor of Arabidopsis cytosolic HSP70 expression at low temperature [14, 45].

Our preliminary explorative findings necessitate more research and call for cautious interpretations. Yet, they indicate that HIBAT can serve as a versatile useful tool for direct screens for heat-sensing and signaling mutants and to identify loss-of-HSR repression mutants.

## Conclusion

Our findings lay the foundation for future research using loss-of-function mutants involved in the heat-signaling, the regulation of HSP-expression, HSP chaperones in particular, and their role in the onset of acquired thermotolerance in plants. The HIBAT reporter line is a powerful and valuable tool to identify by genetic screens new targets involved in plant responses to heat stresses. It may ultimately contribute to the research aiming to ameliorate plant thermotolerance and design of future strategies to mitigate the dramatic adverse effects of global warming on crop plants [6].

## Material and methods

### Generation of HIBAT

#### Transgene design and transformation of *A. thaliana* plants

The pGmHsp17.3b, *nLUC*, and *DAO1* DNA sequences [9, 22, 63] were synthesized by GenScript Biotech (https://www.genscript.com) and cloned into the destination vector pFR7m24GW (provided by Prof. Niko Geldner, University of Lausanne) containing the FastRed cassette for transgenic seed selection [67] and was transformed into *A. thaliana* Col-0 background via *Agrobacterium tumefaciens* (strain GV3101) by floral-dip method [12, 84].

#### Selection of transformant seeds and determination of copy number of HIBAT

The choice for the HIBAT line was based the presence of a single copy of the transgene, as first evidenced by a mendelian segregation in the T2 generation of 1:4 ratio for red transformed seeds over black non-transformed seeds. This was confirmed by whole genome sequencing [62]. Reads were blasted against to the Col-0 *A. thaliana* genome [1] and the single T-DNA insertion was localized at position 26180497 of chromosome 1. Transgene positioning and orientation was confirmed by PCR.

#### Plant materials and growth conditions

Unless otherwise stated all seeds were in the Columbia-0 (Col-0) background. The quadruple HSFA1 KO mutant was provided by Charng YY lab [49]. *A. thaliana* seeds were surface sterilized with 70% ethanol and sowed on ½ MS plate (Duchefa Biochimie, ref P14881.01), and 0.8% (m/v) plant agar (pH 5.8). Following stratification (48 h, 4 °C, in the dark), plates were transferred to a continuous light growth room (22 °C, 100–120 µmol m − 2 s − 1, 60% humidity) or long days condition (16 h light/8 h dark). After two weeks, plants were transferred to soil if necessary.

#### Physiological assays

HIBAT and Col-0 plants were grown in continuous days conditions at 22 °C. Root length was measured using ImageJ [35] software (version 1.53) at 5 and 10 days for a total amount of 80 seedlings. Regarding fresh weight, shoots from 80 seedlings were dissociated from roots and weighted at 9, 13 or 16 days old. Finally, the percentage of germination of the seed in HIBAT and Col-0 lines were determined at 5 and 10 days old with a total amount of 100 seedlings.

#### Heat treatments

Plant seedlings were heated in a growth cabinet chamber (phytotron), incubator (microbial, for and Additional file 5: Fig. S4 and Fig. 2) or a thermoblock (for Fig. 1C). Respective time, intensity of temperature and age of plant used are indicated in figures (See also experimental design Additional file 7: Fig. S6).

#### Acquired thermotolerance assays

HIBAT and Col-0 *A. thaliana* lines were grown on ½ MS medium 1.2% Agar for 14 days in continuous days conditions and plates were imaged before heat treatment. Plants were submitted to heat priming at 36 °C for 2 h followed by 2 h of recovery at 22 °C and then exposed to a noxious heat shock at 45 °C for 45 min. Unprimed plants were exposed to the same noxious HS. Plants were then grown for 7 days at 22 °C and plates were imaged.

#### Iterative heat stresses on d-valine

HIBAT and Col-0 plants were grown on 0 mM, 20 mM, 25 mM, and 30 mM d-valine Agar plates containing ½ MS medium in a growth cabinet chamber under long day condition (16 h light, 8 h dark) at 22 °C. At day 7, a heat program was set up to apply each day for 5 days, two consecutive 2 h heat-shocks at 38 °C interspaced by 2 h at 22 °C. At day 12, plants were allowed to recover for 2 days at 22 °C and were imaged.

#### Other Stress treatments

##### H_2_O_2_, Mannitol, NaCl and FLG22

2 weeks-old seedlings were transferred in an Eppendorf tube of 1.5 mL containing 250 μM H_2_O_2_, 300 mM mannitol, 150 mM of NaCl, or 1 mM of Flagellin 22 (provided by Prof. Philippe Raymond, University of Lausanne) in a final volume of 1 mL of water for 5 h [61]. Seedlings were then exposed for 1 h to the different stressors at the indicated temperatures and following 2 additional hours at 22 °C, nLUC activity was measured in seedling’s total proteins extracts.

##### Cold stress and chilling

2 weeks-old seedlings were transferred to 0.5 mL Eppendorf tube of 1 ml containing water. Cold stress and chilling were applied for 2 h using a thermoblock (4 °C, 12 °C, 16 °C, and 22 °C) or ice (0 °C), and following 2 additional hours at 22 °C, nLUC activity was measured in seedling’s total proteins extracts.

#### Hormonal and radicicol treatments

Abscisic acid (ABA), salicylic acid (SA), Epibrassinolide (EpiBR) and radicicol were stored in various stock solution in DMSO, while jasmonic acid (JA), methyl-jasmonate (JA) and Indole-3-acetic acid (IAA, auxin) were stored in ethanol. 2 weeks-old seedlings were exposed for 1 h at 22 °C followed by 4 h at 31 °C followed by 2 h of post-incubation at 22 °C, except for the specific radicicol experiment where seedlings were exposed to 38 °C.

#### Isolation of soluble protein and Western-Blot Analysis

Plants materials were grinded at RT by a plastic pestle in an Eppendorf tube of 1.5 mL and resuspended with a BAP buffer containing 50 mM Tris–HCL (pH 7.5), 100 mM NaCl, 250 mM mannitol, 5 mM EDTA, 10% (v/v) glycerol, and protease inhibitor cocktail diluted at 1:300 (v/v) (Sigma-Aldrich, ref P9599). Plants extracts were centrifuged for 10 min at 12′000 g at 4 °C. Supernatants containing soluble proteins fraction were transferred in a new Eppendorf tube of 1.5 mL and the total protein concentration was determined by BRADFORD assay (Sigma-Aldrich, ref 23238).

#### Nanoluciferase activity

nLUC-DAO1 bioluminescence was detected by using the Nano-Glo Luciferase Assay System Kit from Promega (ref 1110) containing  furimazine and the HIDEX plate reader (version 5067). The bioluminescence was analyzed for 1-s giving count per second. Using a standard curved with purified nanoluciferase, the bioluminescence emission of total protein extracts was then converted in ng of nLUC-DAO1 per ug total protein in the extract.

##### In vivo observation of nanoLuc activity

The substrate of the nano-luciferase diluted at 1:100 (v/v) [furimazine, Kit from Promega (ref 1110)] was spread on seedling that grew on Agar plates. Light emitting seedlings were imaged using an ImageQuant LAS 500.

#### EMS mutagenesis of HIBAT seeds and screening for mutations

 ~ 6 000 seeds were mutagenized using 0.4% ethyl methanesulfonate (EMS) in 100 mM phosphate buffer, pH 7.5 for 8 h, then washed several times in 100 mM phosphate buffer, pH 7.5 as previously described [44]. M1 seeds were sowed in the soil to generate and harvest the M2 generation of HIBAT mutant lines in a growth chamber with long days conditions (16 h of light, 8 h of dark) at 22 °C. Heat-treated plants showing decreased nanoluciferase levels and non-heated plants showing higher nanoluciferase levels at 22 °C were retained as candidates and their phenotype was further addressed by western blot in the M3 generation. Plants phenotypes were determined by nanoluciferase activity and western blotting.

#### RNA sequencing

2 weeks old HIBAT seedlings were exposed to 22 °C or 38 °C for 40 min. RNA was extracted from plants using MACHEREY–NAGEL NucleoSpin RNA plant KIT (REF 740949.50). Three biological replicates (10–15 seedlings in each replicate) for each condition were sent for RNA sequencing with a total of 1ug of RNA with the Illumina TruSeq Stranded mRNA library- It was then sequenced on the Illumina HiSeq 2500 platform. Purity-filtered reads were adapters and quality trimmed with Cutadapt v. 1.8 [52]. Reads matching to ribosomal RNA sequences were removed with FastQ Screen v. 0.11.1 [80]. Remaining reads were further filtered for low complexity with Reaper v. 15–065 from the Kraken suite [15]. Reads were aligned against *A. thaliana*. TAIR10.39 genome using STAR v. 2.5.3a [19]. The number of read counts per gene locus was summarized with htseq-count v. 0.9.1 [3] using *A. thaliana*. TAIR10.39 gene annotation. Quality of the RNA-seq data alignment was assessed using RSeQC v. 2.3.7 [79]. Reads were also aligned to the *A. thaliana*. TAIR10.39 transcriptome using STAR v. 2.5.3a [19] and the estimation of the isoforms abundance in Transcript Per Million (TPM) was computed using RSEM v. 1.2.31 [48]. Genes with low counts were filtered out. Differential expression was computed with DESEQ2 package [51]. P-values were adjusted by the Benjamini-Hochberg (BH) method, controlling for the false discovery rate (FDR). The RNAseq data set has been deposited to the Sequence Read Archive (SRA) at the National Center for Biotechnology Information (NCBI) repository with the dataset identifier PRJNA948681.

#### Figures and Statistical analyses

Figures and statistical analysis were made using GraphPad Prism version 9.5.0 for Windows, GraphPad Software, San Diego, California USA, www.graphpad.com

## Supplementary Information

**Additional file 1.** Excel file containing raw data for this study.

**Additional file 2.: Figure S1.** Translated protein sequence of the transgene. Red: nLUC derived from the catalytic subunit of oplophorus-luciferin 2-monooxygenase found in Oplophorus gracilirostris. Blue: DAO-1from Rhodosporidium toruloides. Black: Linker between DAO and nLUC and a C-terminal epitope for Flag antibodies [43]. Full protein and gene sequences in (Additional file 1: Tab 12).

**Additional file 3: Figure S2.  **Scheme of the transgene. LB refers to the left border of the T-DNA insertion site. The promoter of OLEOSIN 1has been previously described by Zhong et al. [85]. Oleo RFP-TAG represents the constitutive expression of a red fluorescent proteinfusion tag coupled with OLEOSIN 1. The soybean heat-inducible promoterhas been reported by Treuter et al. [75]. The construct nLUC-DAO1+Flag contains the nLUC gene, a novel and versatile small bioluminescence platform described by England et al. [21], fused with d-amino acid oxidaseand an added Flag epitope. DAO1 acts as a conditionally toxic negative marker in the presence of d-valine, as previously reported by Gisby et al. [28].

**Additional file 4: Figure S3.**  Physiological assessments to compare the performance of HIBAT and Col-0 lines**.** **A** Shoot fresh weight was measured at 9, 13, and 16 days of age for both HIBAT and Col-0 lines, with 50 seedlings analyzed per line. **B** Root length was measured at 5 and 10 days of age for HIBAT and Col-0 plants, with 80 seedlings analyzed per line. **C** The germination percentage of seeds was determined at 5 and 10 days of age for HIBAT and Col-0 lines, with 100 seedlings analyzed per line. Statistical analysis using a 2-way ANOVA was performed to identify significant differences, denoted by asterisks, while "NS" indicates no significant difference.

**Additional file 5: Figure S4.**  Acquired thermotolerance assay in the HIBAT and Col-0 lines. Left: no priming, right: with priming. Conditions applied for each plant are represented on top. Pictures were imaged at 14 and 21 days old. Percentage of dead cotyledons but surviving plants: around 46% in Col-0 and 76% in HIBAT.

**Additional file 6: Figure S5.** Expression of nLUC-DAO1, HSP17.7 and HSP101 in HIBAT. Left: The levels of accumulated nLUC-DAO in two-week-old HIBAT seedlings were quantified based on the relative nanoluciferase activity in crude extracts. The seedlings were pretreated for 1 hour at either 22 °C or 38 °C. Center and Right: Immunodetection was performed to assess the expression of Arabidopsis HSP17.7 and HSP101 in HIBAT seedlings, with or without a one-hour pretreatment at 38 °C.

**Additional file 7: Figure S6.** Experimental design for iterative heat stress.

**Additional file 8: Figure S7.** Quantification of HSP17.7 and HSP101 accumulation in selected M3 HIBAT mutants under heat shock conditions. The percentage of HSP accumulation was determined through Western blot analysis of M3 HIBAT candidate mutants, with normalization against the intensity of HSP signal observed in the HIBAT line after heat shock treatment. The quantification of HSP accumulation was further normalized using RUBISCO expression levels in the respective samples. ImageJ software was employed for the quantification process. [35].

**Additional file 9: Figure S8.** The L5 mutant exhibits hyperthermosensitivity. **A** Representative images of control pre-heat treated HIBAT seedlingand L5 mutant. **B** Five-week-old leaves from the parental HIBAT line and the L5 mutant were subjected to different temperaturesfor 2 hours followed by 2 hours of post-recovery at 22 °C to test nLUC expression. **C** Western blot analysis showing the expression levels of sHSP17.7 and HSP101. The loading control for each protein sample is visualized by Coomassie blue staining of RubisCO. Asterisks indicate statistically significant differences determined by Student t-test.

## Data Availability

The data underlying this article are available in the article and in its online supplementary material.
