# Supplementary figures and images for: Design of an Arabidopsis thaliana reporter line to detect heat-sensing and signaling mutants
Source: Plant Methods. 2023 Jun 8;19:56. doi: 10.1186/s13007-023-01033-x (PMC10251684; doi:10.1186/s13007-023-01033-x)

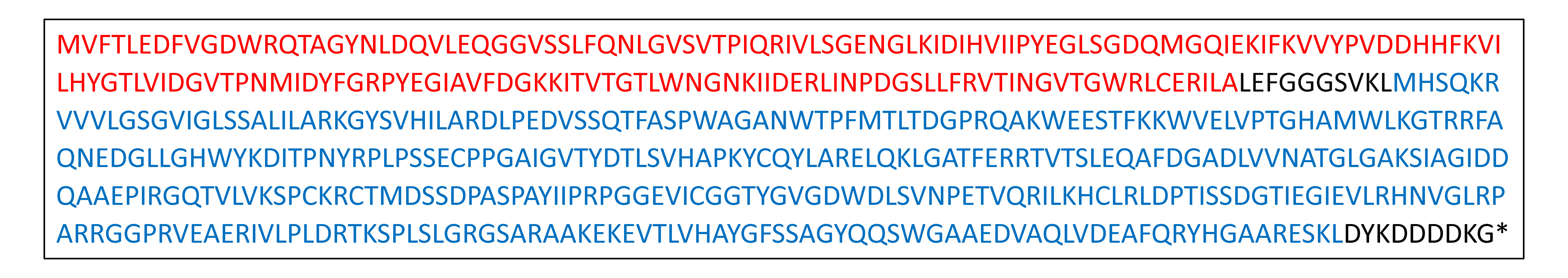

Supplement: Supplementary file 2 — Additional file 2.: Figure S1. Translated protein sequence of the transgene. Red: nLUC derived from the catalytic subunit of oplophorus-luciferin 2-monooxygenase found in Oplophorus gracilirostris. Blue: DAO-1from Rhodosporidium toruloides. Black: Linker between DAO and nLUC and a C-terminal epitope for Flag antibodies [43]. Full protein and gene sequences in (Additional file 1: Tab 12). [file 13007_2023_1033_MOESM2_ESM.jpg]

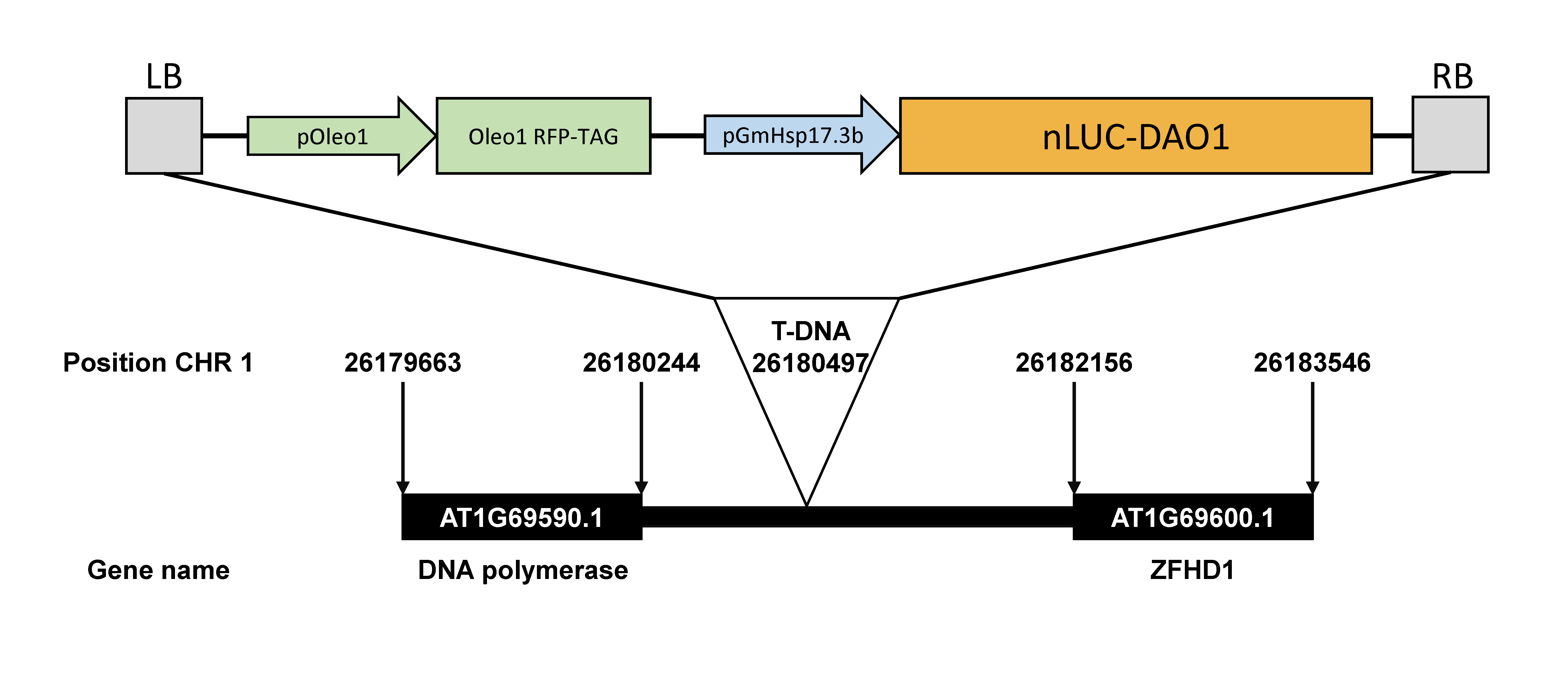

Supplement: Supplementary file 3 — Additional file 3: Figure S2. Scheme of the transgene. LB refers to the left border of the T-DNA insertion site. The promoter of OLEOSIN 1has been previously described by Zhong et al. [85]. Oleo RFP-TAG represents the constitutive expression of a red fluorescent proteinfusion tag coupled with OLEOSIN 1. The soybean heat-inducible promoterhas been reported by Treuter et al. [75]. The construct nLUC-DAO1+Flag contains the nLUC gene, a novel and versatile small bioluminescence platform described by England et al. [21], fused with d-amino acid oxidaseand an added Flag epitope. DAO1 acts as a conditionally toxic negative marker in the presence of d-valine, as previously reported by Gisby et al. [28]. [file 13007_2023_1033_MOESM3_ESM.jpg]

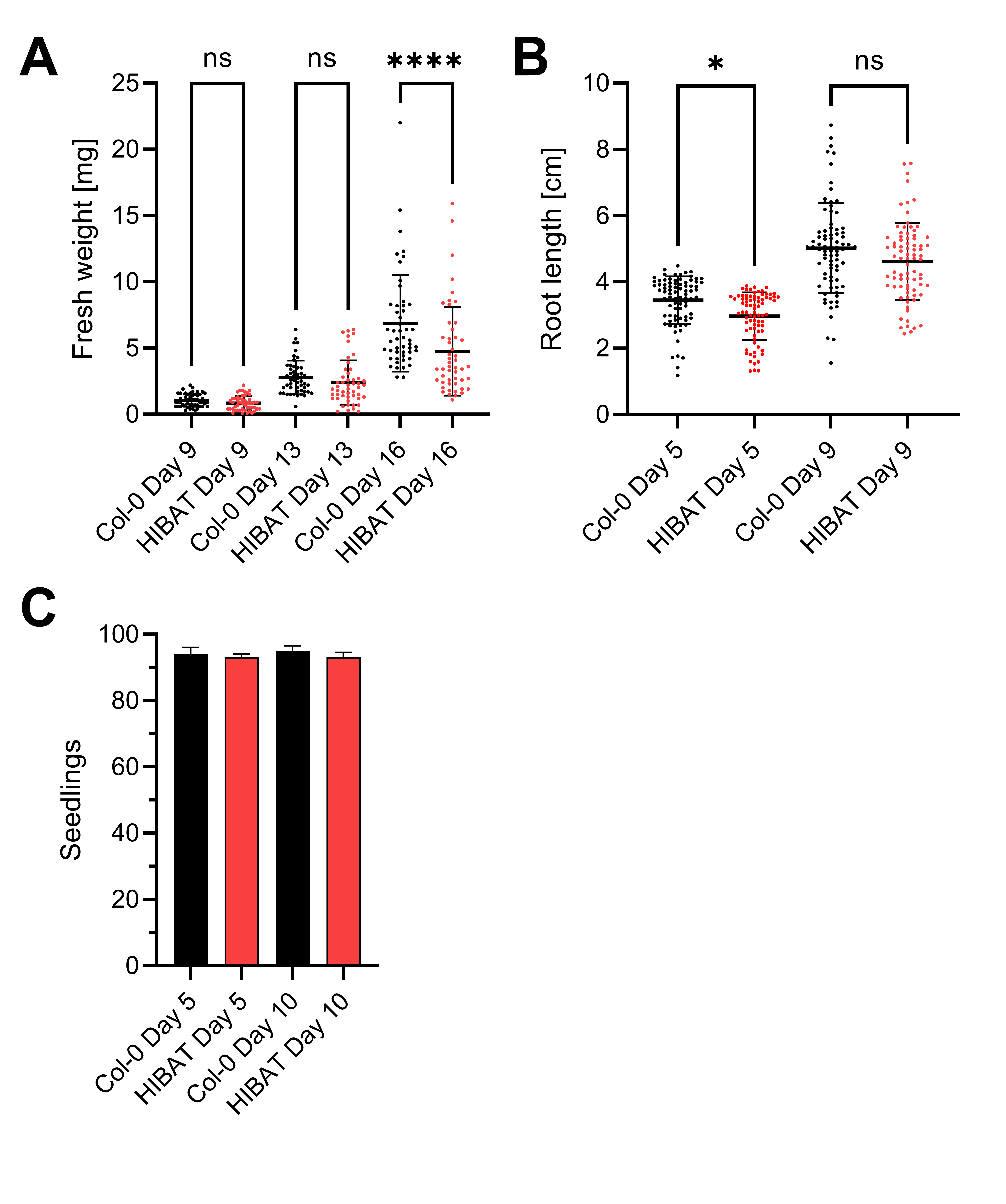

Supplement: Supplementary file 4 — Additional file 4: Figure S3. Physiological assessments to compare the performance of HIBAT and Col-0 lines. A Shoot fresh weight was measured at 9, 13, and 16 days of age for both HIBAT and Col-0 lines, with 50 seedlings analyzed per line. B Root length was measured at 5 and 10 days of age for HIBAT and Col-0 plants, with 80 seedlings analyzed per line. C The germination percentage of seeds was determined at 5 and 10 days of age for HIBAT and Col-0 lines, with 100 seedlings analyzed per line. Statistical analysis using a 2-way ANOVA was performed to identify significant differences, denoted by asterisks, while "NS" indicates no significant difference. [file 13007_2023_1033_MOESM4_ESM.png]

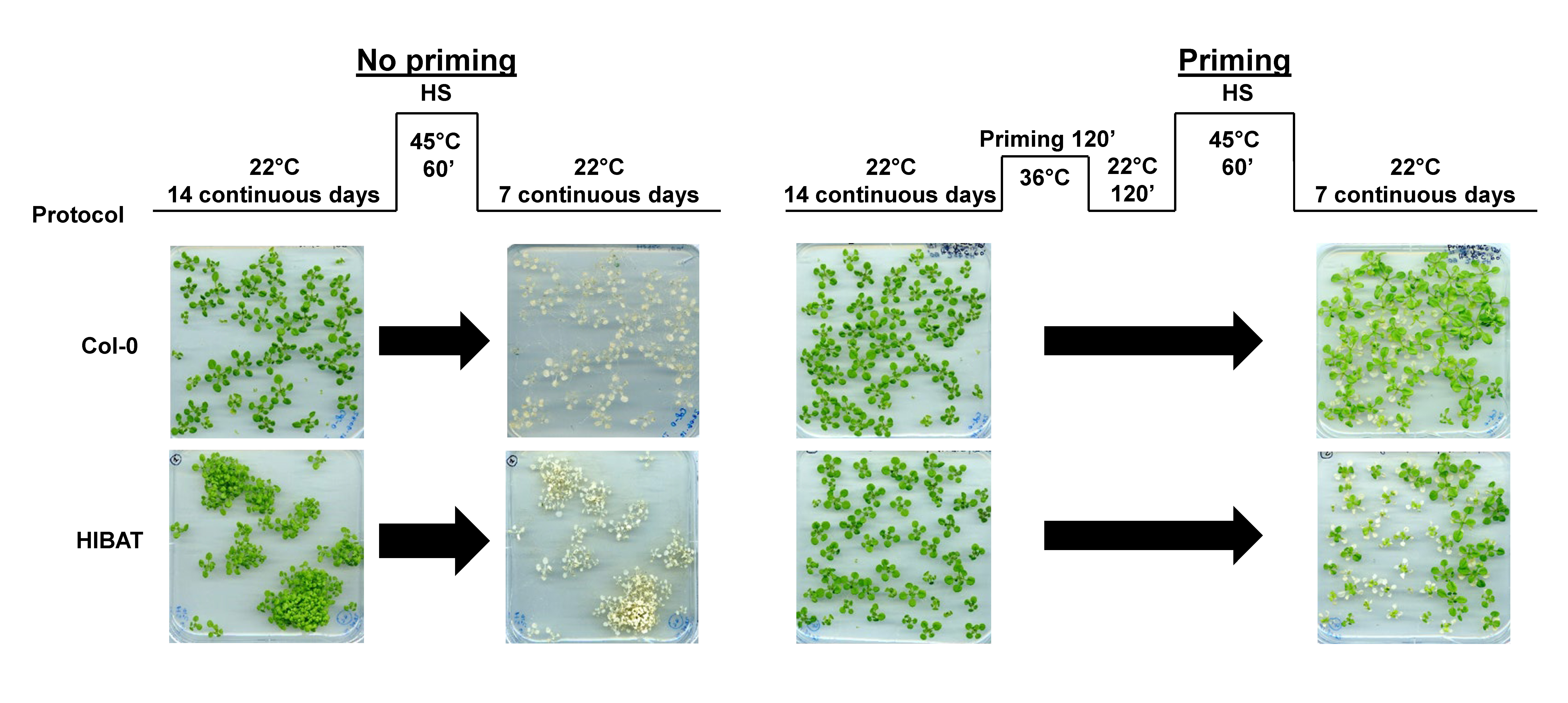

Supplement: Supplementary file 5 — Additional file 5: Figure S4. Acquired thermotolerance assay in the HIBAT and Col-0 lines. Left: no priming, right: with priming. Conditions applied for each plant are represented on top. Pictures were imaged at 14 and 21 days old. Percentage of dead cotyledons but surviving plants: around 46% in Col-0 and 76% in HIBAT. [file 13007_2023_1033_MOESM5_ESM.jpg]

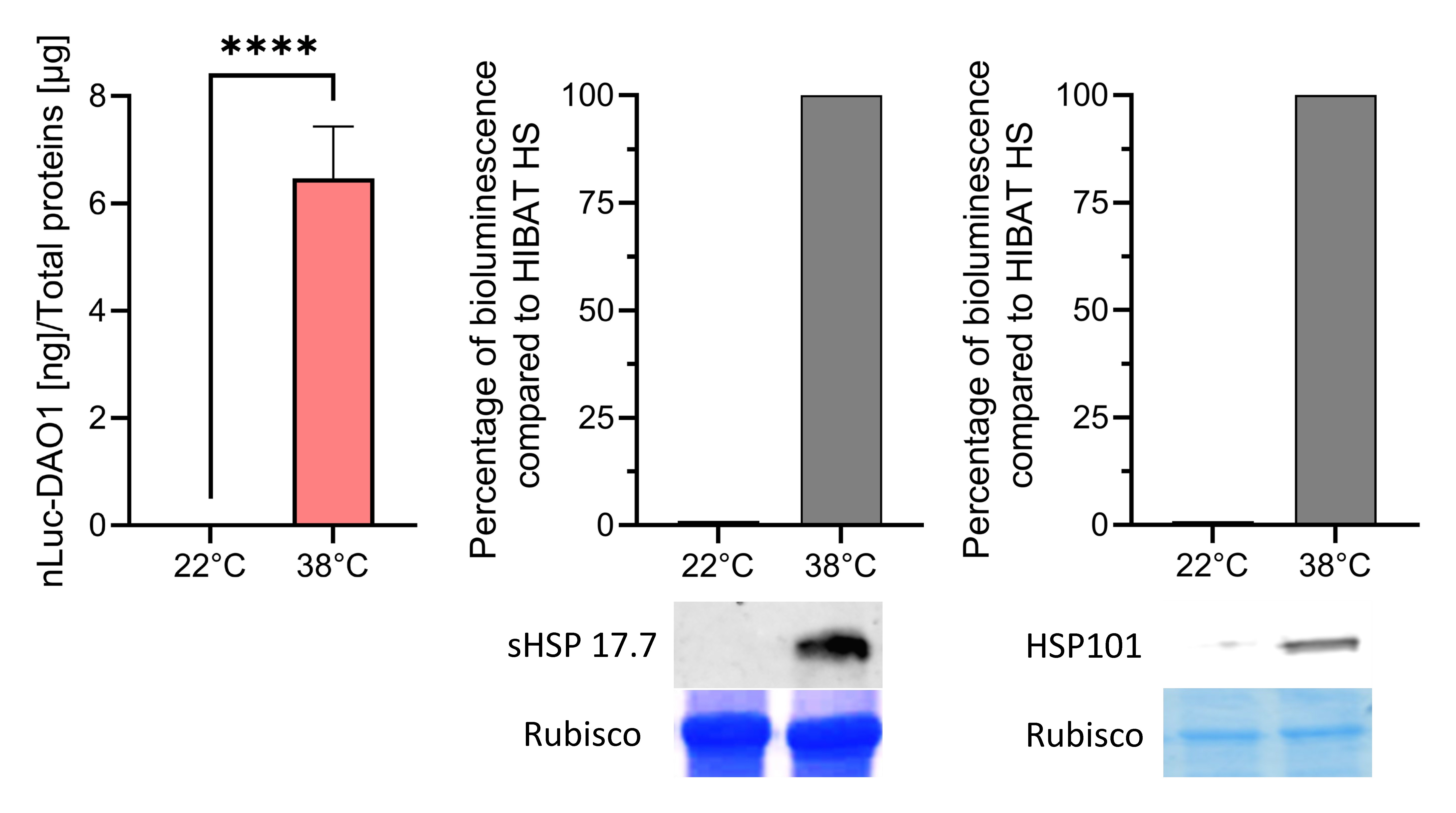

Supplement: Supplementary file 6 — Additional file 6: Figure S5. Expression of nLUC-DAO1, HSP17.7 and HSP101 in HIBAT. Left: The levels of accumulated nLUC-DAO in two-week-old HIBAT seedlings were quantified based on the relative nanoluciferase activity in crude extracts. The seedlings were pretreated for 1 hour at either 22 °C or 38 °C. Center and Right: Immunodetection was performed to assess the expression of Arabidopsis HSP17.7 and HSP101 in HIBAT seedlings, with or without a one-hour pretreatment at 38 °C. [file 13007_2023_1033_MOESM6_ESM.jpg]

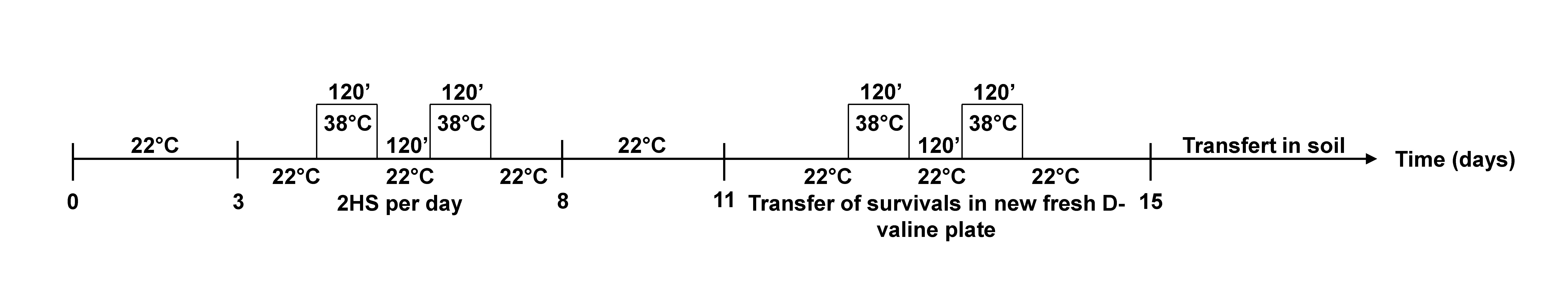

Supplement: Supplementary file 7 — Additional file 7: Figure S6. Experimental design for iterative heat stress. [file 13007_2023_1033_MOESM7_ESM.jpg]

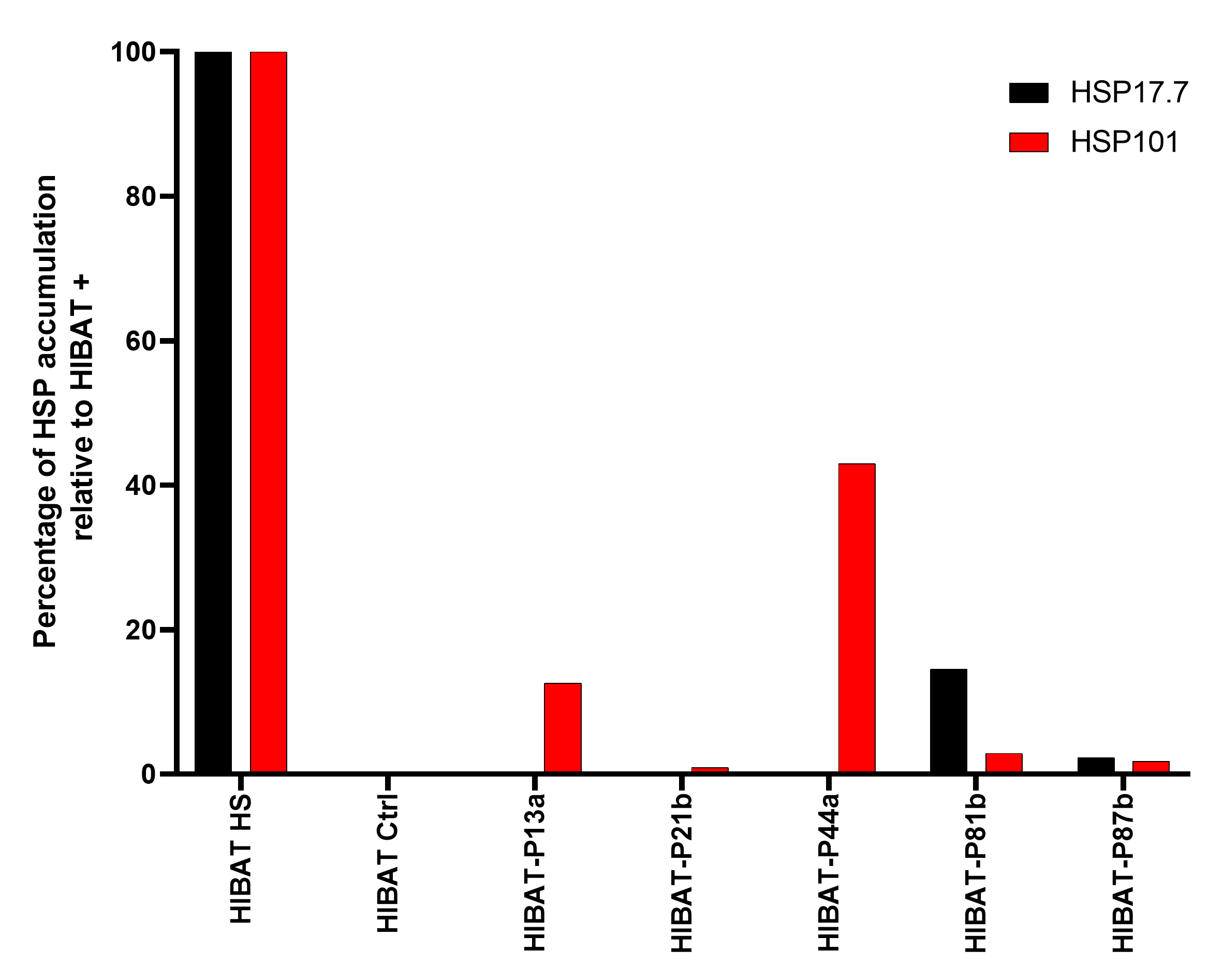

Supplement: Supplementary file 8 — Additional file 8: Figure S7. Quantification of HSP17.7 and HSP101 accumulation in selected M3 HIBAT mutants under heat shock conditions. The percentage of HSP accumulation was determined through Western blot analysis of M3 HIBAT candidate mutants, with normalization against the intensity of HSP signal observed in the HIBAT line after heat shock treatment. The quantification of HSP accumulation was further normalized using RUBISCO expression levels in the respective samples. ImageJ software was employed for the quantification process. [35]. [file 13007_2023_1033_MOESM8_ESM.png]

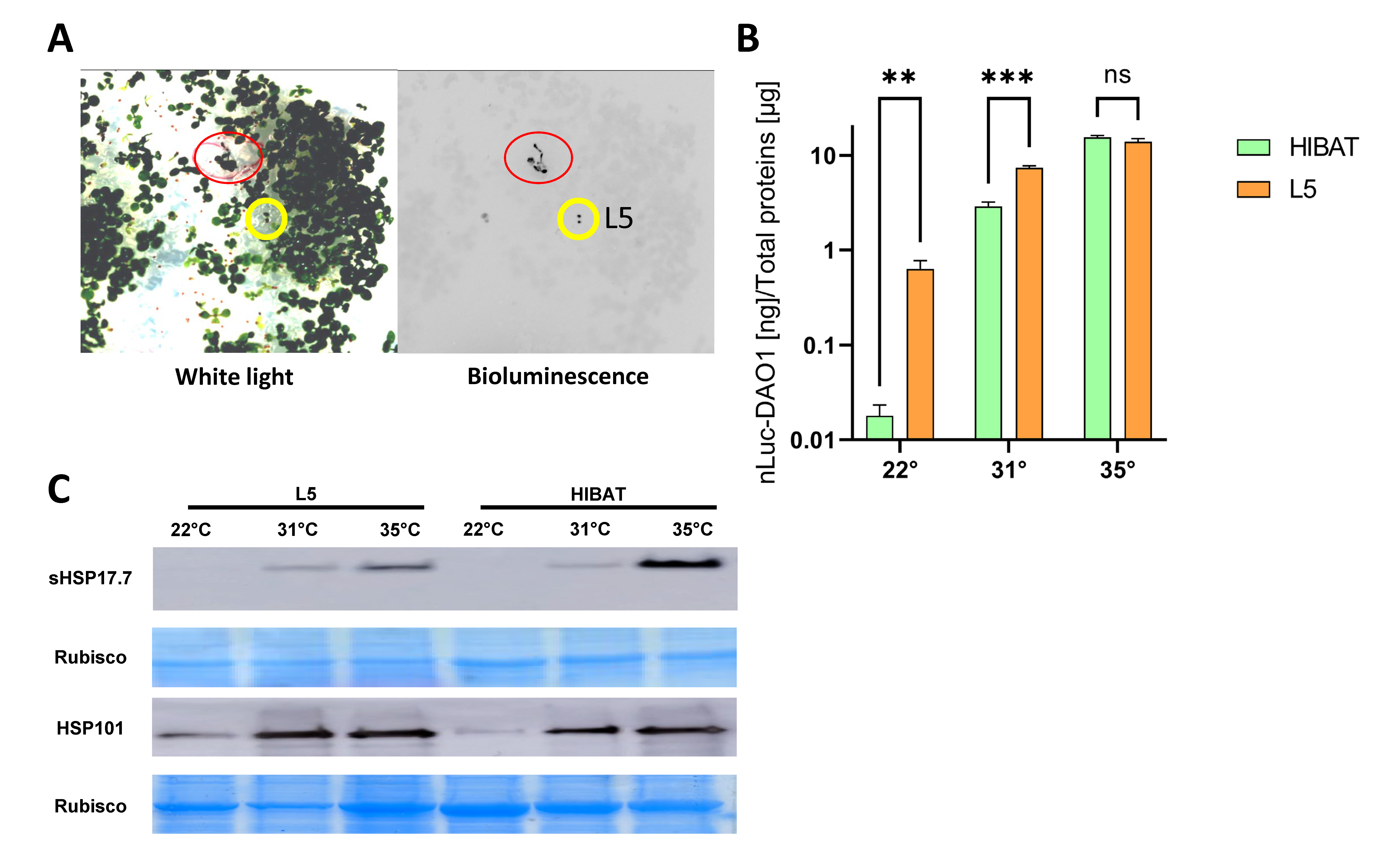

Supplement: Supplementary file 9 — Additional file 9: Figure S8. The L5 mutant exhibits hyperthermosensitivity. A Representative images of control pre-heat treated HIBAT seedlingand L5 mutant. B Five-week-old leaves from the parental HIBAT line and the L5 mutant were subjected to different temperaturesfor 2 hours followed by 2 hours of post-recovery at 22 °C to test nLUC expression. C Western blot analysis showing the expression levels of sHSP17.7 and HSP101. The loading control for each protein sample is visualized by Coomassie blue staining of RubisCO. Asterisks indicate statistically significant differences determined by Student t-test. [file 13007_2023_1033_MOESM9_ESM.jpg]
